# Isoindoline‐Based Nitroxides as Bioresistant Spin Labels for Protein Labeling through Cysteines and Alkyne‐Bearing Noncanonical Amino Acids

**DOI:** 10.1002/cbic.201900537

**Published:** 2019-12-06

**Authors:** Theresa Sophie Braun, Pia Widder, Uwe Osswald, Lina Groß, Lara Williams, Moritz Schmidt, Irina Helmle, Daniel Summerer, Malte Drescher

**Affiliations:** ^1^ Department of Chemistry University of Konstanz Universitätsstrasse 10 78457 Konstanz Germany; ^2^ Konstanz Research School Chemical Biology (KoRS-CB) University of Konstanz Universitätsstrasse 10 78457 Konstanz Germany; ^3^ Faculty of Chemistry and Chemical Biology TU Dortmund Otto-Hahn-Strasse 4a 44227 Dortmund Germany; ^4^ Present address: Faculty of Science Department of Pharmaceutical Biology University of Tübingen Auf der Morgenstelle 8 72076 Tübingen Germany

**Keywords:** bioresistance, EPR spectroscopy, macromolecular dynamics, protein conformation analysis, site-directed spin labeling

## Abstract

Electron paramagnetic resonance (EPR) spectroscopy in combination with site‐directed spin labeling (SDSL) is a powerful tool in protein structural research. Nitroxides are highly suitable spin labeling reagents, but suffer from limited stability, particularly in the cellular environment. Herein we present the synthesis of a maleimide‐ and an azide‐modified tetraethyl‐shielded isoindoline‐based nitroxide (M‐ and Az‐TEIO) for labeling of cysteines or the noncanonical amino acid *para*‐ethynyl‐l‐phenylalanine (*p*ENF). We demonstrate the high stability of TEIO site‐specifically attached to the protein thioredoxin (TRX) against reduction in prokaryotic and eukaryotic environments, and conduct double electron–electron resonance (DEER) measurements. We further generate a rotamer library for the new residue *p*ENF‐Az‐TEIO that affords a distance distribution that is in agreement with the measured distribution.

## Graphical abstract

**DEER in the distance**: Isoindoline‐based nitroxides for site‐directed spin labeling of proteins show a good half‐life in bacterial and eukaryotic cell extracts. Distance distributions revealed from double electron–electron resonance (DEER) measurements between two labeling sites in thioredoxin are in good agreement with the simulated distribution using a rotamer library.

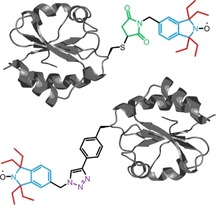

Electron paramagnetic resonance (EPR) spectroscopy is a powerful tool to investigate protein structure and dynamics. In particular, double electron–electron resonance (DEER) technique^1^ enables revealing distance distributions in the nanometer range.^2^ Therefore, the pairwise introduction of spin labels by site‐directed spin labeling (SDSL) is required.^3^ SDSL is based on chemical selective and stable linkage of an EPR‐active species to the target. Spin labeled biological macromolecules can be studied by DEER even in the context of their native, cellular environment,^4^ as biological organisms own a limited amount of endogenous EPR‐active species, for example, manganese, copper and iron ions. Endogenous paramagnetic species are spectrally distinguishable from typical spin labels such as lanthanides,^5^ copper,^6^ trityl,^7^ or—the most commonly employed spin labels^8^—nitroxides. Owing to their small size, nitroxides are well tolerated by proteins, feature high sensitivity due to their narrow EPR spectrum, and enable monitoring dynamics of the labeled protein because their spectral shape depends on rotational diffusion.

There are two key features for in cell applicability of nitroxide based spin labels: 1) the stability of the nitroxide head group against intracellular reduction, which is known to be a limiting factor in biological surroundings^4^, ^9^ and 2) the attachment strategy.

Radical stability against reduction is determined by the affiliated ring structure of the nitroxide moiety and steric shielding by α‐substituents.^10^ For attachment strategies several aspects have to be considered: labeling efficiency, nontoxicity, stable attachment, and bio‐orthogonality, that is, exclusion of off‐target labeling.

Here, we report the synthesis, characterization and application of nitroxide labels that are based on an isoindoline ring equipped with four ethyl groups in α‐position to the nitroxide (1,1,3,3‐**t**etra**e**thyl**i**soindolin‐2‐yl**o**xyl10a, 11), for which Marx et al. introduced the acronym TEIO.10a, 12 We modify TEIO either with a maleimide (M‐TEIO, Figure 1 A) or an azide function (Az‐TEIO, Figure 1 B) and demonstrate the resistance of TEIO against nitroxide reduction in prokaryotic as well as eukaryotic environments.

**Figure 1:**
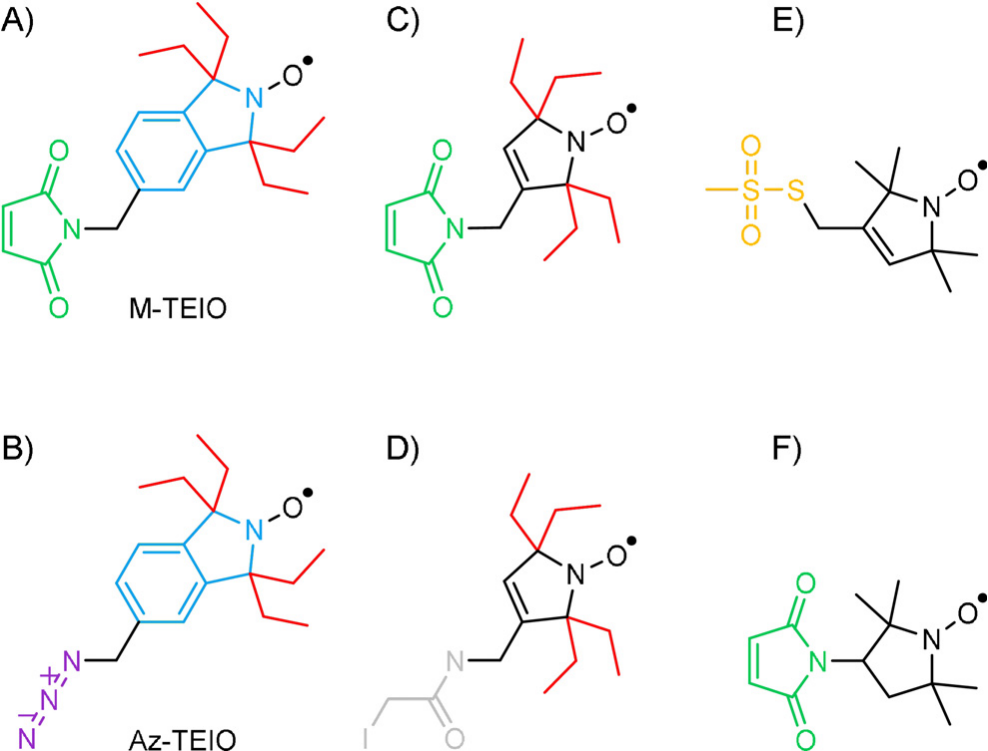
Nitroxide labeling reagents discussed in this work: A) M‐TEIO, B) Az‐TEIO, C) M‐TETPO/MAG, D) IAG, E) MTSL, and F) M‐Proxyl. They can be grouped according to the nitroxide ring structure, the α‐substituents, and the chemical moiety that is used for linkage in SDSL. Examined ring structures include pyrrolidines and isoindolines (blue), α‐substituents were either methyl or ethyl (red) groups. Depending on the SDSL strategy (Figure S8), either thiosulfonate ester (yellow), maleimide (green), iodoacetamide (grey), or azide (purple) functionality was applied.

Nitroxide‐based spin labels with particularly high stability against reduction have been developed that are based on tetraethyl‐group shielded pyrrolidines (Figure 1 C, D). They have been applied to protein SDSL as maleimides or as iodoacetamides for reacting cysteines via Michael addition or iodide substitution.5c, 13 In addition to such shielding effects by α‐substituents, it is known that nitroxides are more stable if they are embedded in pyrrolidines (Figure 1 E, F) as compared to piperidines.^14^ Spin labels based on isoindolines (Figure 1 A, B) have the potential to exhibit even higher stability than pyrrolidine‐based spin labels.10b, 10e Isoindoline‐based nitroxides have been used for RNA labeling5d, 15 and an improved stability was shown for tetraethyl‐shielded relative to tetramethyl‐shielded isoindoline‐labels.^16^ A methyl‐shielded isoindoline‐based nitroxide was applied to tyrosine labeling, and used for probing protein‐structural change.^17^

*S*‐(1‐Oxyl‐2,2,5,5‐tetramethyl‐2,5‐dihydro‐1*H*‐pyrrol‐3‐yl)methyl methanesulfonothioate (MTSL, Figure 1 E) coupling to cysteine residues results in the standard EPR‐active side chain R^1^,^3^, ^18^ but its inherent disulfide bond suffers from low stability in biological environments.^19^ Michael addition of maleimides (Figure 1 A, C, F) to cysteines forms stable C−S bonds, but due to off‐target labeling of proteins bearing endogenous cysteines, it cannot be performed directly in cells. The use of genetically encoded noncanonical amino acids (ncAA) for SDSL promises bio‐orthogonality including excellent chemoselectivity and potential for experiments in complex biological environments such as living cells.^20^ Besides condensation reactions with ketone amino acids,^21^ strain‐promoted azide–alkyne cycloadditions (SPAAC),^22^ Suzuki coupling,^23^ strain‐promoted inverse‐electron‐demand Diels–Alder reaction (SPIEDAC),^24^ and direct encoded spin labeled amino acids,9c, 25 copper(I)‐catalyzed azide–alkyne cycloaddition (CuAAC)22b, 26 has been shown to be suitable for SDSL with nitroxides.

In the current work, we synthesize TEIO variants (Scheme 1) and use them as nitroxide label. The synthesis started from amine **1** previously reported from Bottle and co‐workers.^11^ The benzylic position was oxidized by KMnO_4_ to the corresponding carboxylic acid **2**. Reduction with LiAlH_4_ yielded alcohol **3**, which was deprotected by catalytic hydrogenation to yield secondary amine **4**. Subsequent oxidation with *m*CPBA afforded nitroxide **5** which was functionalized by conversion into mesylate **6** and substitution with NaN_3_ to Az‐TEIO (**7**). For the introduction of the maleimide functionality, Staudinger reaction was performed to yield benzylic amine **8** which was then converted into M‐TEIO (**9**) in a two‐step procedure using maleic anhydride. Further details can be found in the Supporting Information.

**Scheme 1:**
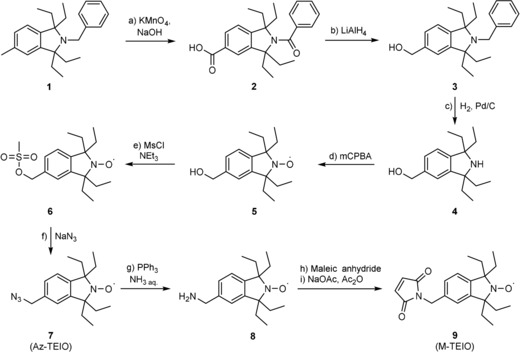
Synthesis of Az‐TEIO (**7**) and M‐TEIO (**9**): a) KMnO_4_, NaOH/pyridine, H_2_O, 120 °C, 48 h, 80 %; b) LiAlH_4_/THF, 0 °C, 48 h, 99 %; c) H_2_, Pd/C/AcOH, overnight, 47 %; d) *m*CPBA/CH_2_Cl_2_, 0 °C, 20 h, 76 %; e) NEt_3_, MsCl/CH_2_Cl_2_, 0 °C, 3 h; f) NaN_3_/DMF, 20 h, 79 %; g) PPh_3_/THF, 0 °C, 30 min, NH_3_(25 %)/THF, 0 °C, 21 h, quant.; h) maleic anhydride/THF, RT, 50 min; i) NaOAc/Ac_2_O, 74 °C, 1.5 h, 64 % over two steps.

For SDSL with M‐TEIO, we used site‐directed mutagenesis to remove the two endogenous cysteines in the oxidoreductase enzyme thioredoxin (TRX) and introduced new cysteines at the desired labeling positions. TRX variants containing either two (TRX D14C R74C, TRX D14C G34C) or no cysteines (TRX WT*) were expressed, purified, and subjected to the labeling protocol (Figure S8 in the Supporting Information). Spin labeling using M‐TEIO was selective for cysteine containing TRX variants (Figure S9). We observed partial dimerization of cysteine variants, which was completely suppressed by addition of tris‐(2‐carboxyethyl)phosphine hydrochloride (TCEP) to the labeling reaction mixture. Labeling efficiency was determined by the quotient of spin concentration and labeling sites, and was found to be ≈70 %.

M‐TEIO stability against chemical reduction was tested by following the reduction profile of 200 μm spin label in presence of 4 mm sodium ascorbate in analogy to the corresponding experiment described for ethyl‐shielded pyrrolidine (Figure 1 C, M‐TETPO5c), and compared with M‐Proxyl^27^ as representative for standard labeling reagents based on methyl‐shielded pyrrolidines (Figure 2 F). After 19 minutes, only 50 % of the EPR signal of M‐Proxyl was left, whereas no change in signal intensity of M‐TEIO was detected. M‐TETPO intensity has been reported to be decayed to 90 %5c after 60 minutes under the same conditions. Even after 70 h, when no signal of M‐Proxyl was detectable anymore, 60 % of the M‐TEIO signal was still present. (Figure 2 A). We also tested the stability of Az‐TEIO in ascorbate, which did not show any loss of EPR signal within 16 h (Figure S10). It is known that nitroxide label stability depends on the cell type.^13^ Depending on the target protein, spin labels are required to be stable in different cell types. Therefore, we exposed the nitroxides to two different cell types. Prokaryotic *Escherichia coli* and eukaryotic HEK lysates were prepared freshly and mixed with TRX D14C R74C labeled either with M‐TEIO or M‐Proxyl. We did not observe any change of the spectral shape of the M‐TEIO signal during the experiment, and the spectral shape was similar to that in solution (Figure S11). The half‐lives of M‐TEIO (*E. coli*: 6.1 h, HEK: 3.3 h) were found to be four‐ and sixfold higher than M‐Proxyl (*E. coli*: 1.5 h, HEK: 35 min; Figure 3 B, C). Hence, M‐TEIO opens the door to investigate proteins either in these prokaryotic or eukaryotic host cells.

**Figure 2:**
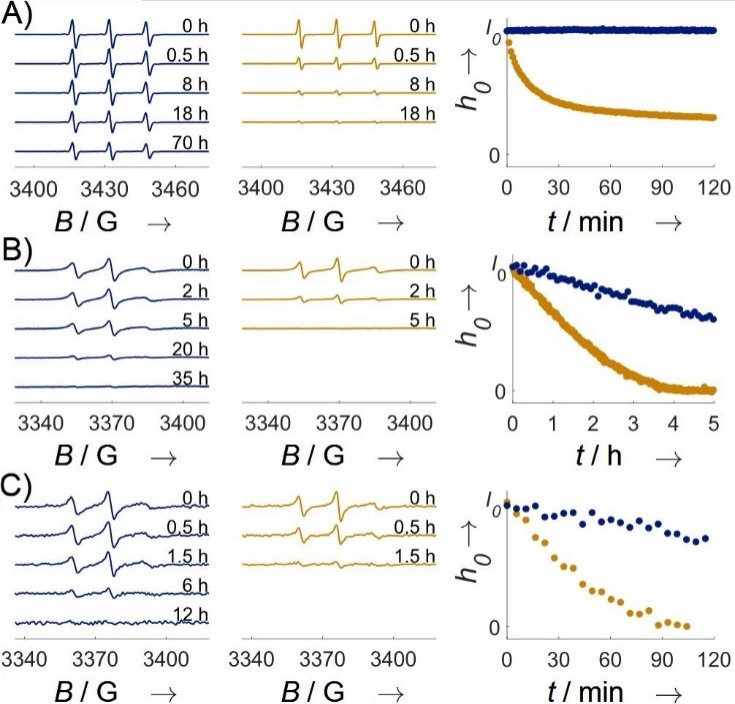
Reduction stability of M‐TEIO (blue) compared with M‐Proxyl. A) Reduction of label in solution with 4 mm ascorbate, B) labeled TRX D14C R74C in *E. coli* lysate, and C) labeled TRX D14C R74C in HEK lysate. Nitroxide signal reduction was measured by time‐resolved CW EPR spectroscopy. Peak‐to‐peak intensity was used as a measure for nitroxide stability and set to *I*_0_ for *t*=0 h, which corresponds to a nitroxide concentration of 200 μm in ascorbate mix, 30 μm in *E. coli* and 6 μm in HEK lysate.

**Figure 3:**
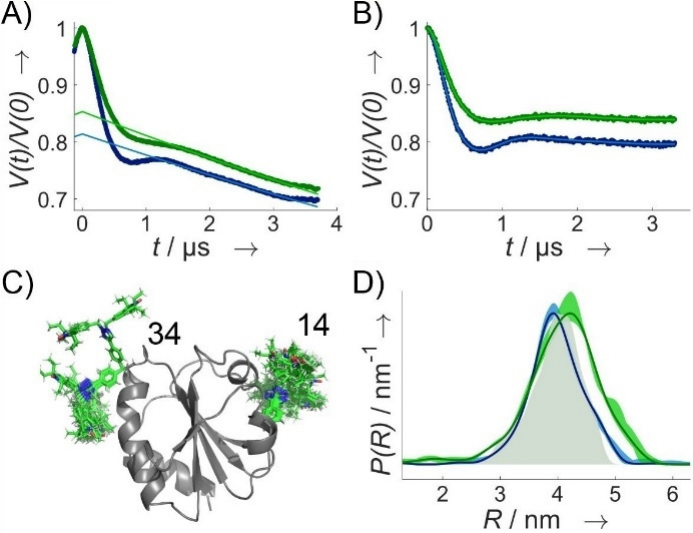
DEER data for TRX doubly labeled with M‐TEIO (blue, *c*_spin_=50 μm, *c*_protein_=34 μm) or Az‐TEIO (green, *c*_spin_=108 μm, *c*_protein_=90 μm) measured at Q‐Band at 50 K. A) DEER traces. B) Form factor obtained after 3D background correction. C) Crystal structure of TRX (PDB ID: https://www.rcsb.org/structure/2TRX)^31^ with attached rotamers for Az‐TEIO at *p*ENF at position 14 and 34 of the primary protein structure. D) Distance distribution predicted using the rotamer library for Az‐TEIO (grey shaded areas), experimental distance distribution including validation.

To test the capability of M‐TEIO for obtaining long range distance restraints by DEER, we applied the M‐TEIO labeling protocol to TRX D14C G34C and performed DEER (Figures 3 and S12). Because cysteine labeling in general is not bio‐orthogonal and therefore M‐TEIO is not suitable for in vivo labeling or for labeling proteins bearing off‐target cysteines, we tested an SDSL‐strategy based on attaching an azide‐modified TEIO (Az‐TEIO, Figure 1 B, **7**) to the genetically encoded, noncanonical amino acid^28^ *para*‐ethynyl‐l‐phenylalanine (*p*ENF)^29^ by click chemistry. We incorporated two *p*ENF into TRX by expression with amber suppression (polyspecific *Methanocaldococcus jannaschii* tRNA^Tyr^(CUA)/tyrosyl‐tRNA‐synthetase (YRS) pair)^30^ at sites D14TAG G34TAG, and labeled the purified protein with Az‐TEIO via copper(I)‐catalyzed azide–alkyne cycloaddition (CuAAC) in analogy to Widder et al.^26^ (Figure S8 B). Although it would have been possible to use a TRX variant that still contains the endogenous cysteines of its catalytic center,9c we chose the cysteine‐free variant for direct comparison with the M‐TEIO results. A labeling efficiency of 60 % was achieved. This nonstoichiometric labeling results in decreased modulation depth in DEER. However, we succeeded in determining a DEER distance distribution (Figure 3). In general there is a toolbox based on artificial amino acids and copper click chemistry enabling full stoichiometric spin labeling.^26^

To interpret the distance distributions obtained by DEER, the flexibility and the length of the linker between paramagnetic center and protein backbone must be taken into account. We thus simulated the contribution of the linker using a rotamer library approach^32^ for *p*ENF‐TEIO. We identified five relevant torsion angles (Figure S13 A) and generated a conformer ensemble by random variation. Based on their energy in a universal force field,^33^ representative angles were selected and clustered resulting in a population‐weighted, averaged set of dihedral angles representing the ensemble (Figure S13 B). The obtained rotamers were attached to the crystal structure of TRX^31^ and simulated (Figure 3 C). The simulated mean distance between the labeling sites (*R*(*P*_max_)=4.0 nm) was in good agreement with the experimental data (*R*(*P*_max_)=4.2 nm; Figure 3 D). The rotamer library will also be helpful when transferring the labeling approach to new biological research questions. However, when investigating proteins of unknown structure, where the rotamer space may be restricted by steric constraints of the protein, the approach might be limited.

Although M‐TEIO does not depend on the incorporation of ncAA, which is interesting for proteins with low expression yield, the Az‐TEIO approach turned out to be an excellent choice regarding biocompatibility and chemoselectivity. CuAAC has been shown to be a suitable chemical reaction for in cell spin labeling.22b Therefore, Az‐TEIO might open the door for in cell SDSL with the potential to perform expression, labeling and investigation in the native environment of the target protein.

In conclusion, we presented two tetraethyl‐shielded isoindoline‐based nitroxides (TEIO) for SDSL and demonstrated that Az‐ and M‐TEIO are even more stable against chemical reduction with ascorbic acid than tetraethyl‐group shielded pyrroline nitroxide spin‐labels. We labeled cysteine introduced site‐specifically into TRX with M‐TEIO and found half‐lives of TEIO to be up to sixfold higher than M‐Proxyl in cell lysates. Our data suggests that in contrast to ethyl‐shielded pyrrolidines, TEIO is suitable for both, prokaryotic and eukaryotic environments. Az‐TEIO enables double labeling via genetically encoded ncAAs, aiming toward in vivo labeling and distance measurements. We provide a rotamer library taking the cell stable tether of Az‐TEIO into account. The chemical and spectroscopic properties of the TEIO‐labels enable applications in EPR experiments combined with SDSL in vitro and have the potential to be used for spectroscopy in cells.

## Experimental Section

Details on TEIO synthesis, TRX expression and purification, labeling and EPR measurements are given in the Supporting Information. Briefly, TRX variants containing a His tag were expressed in *E. coli* and purified with the help of Ni‐NTA beads.

Maleimide coupling of the protein was performed in the presence of a tenfold excess of M‐TEIO at pH 7.4 and 4 °C for 1 to 4 days. TCEP was added to avoid disulfide bond formation. Unbound label reagent was removed using a combination of Ni‐NTA‐chromatography, size‐exclusion chromatography via spin desalting columns (PD‐10), and ultrafiltration in centrifugal filter units.

For copper click reactions, Cu^II^ in complex with the ligand BTTAA was reduced with ascorbic acid into the catalytically active Cu^I^ species. Labeling was performed with a 20‐fold excess of Az‐TEIO in presence of the catalytic mixture that corresponds to a Cu^II^ start concentration of a 20‐fold excess. Excess reagents were removed by size‐exclusion chromatography via spin desalting columns (Zeba^TM^). Ultrafiltration in centrifugal filter units was performed to remove excess reagents, concentrate the protein sample, and exchange the buffer.

Spin concentrations of the labeled protein were determined with the help of an EMXnano spectrometer and the spin‐counting application of the Xepr software (both Bruker Biospin). Labeling efficiencies were determined as the quotient of the spin concentration and the concentration of labeling sites expected from protein concentration.

DEER experiments were performed using a four‐pulse sequence (*π*/2_obs_—*τ*_1_—*π*_obs‐_—*t′*—*π*_pump_—(*τ*_1_+*τ*_2_−*t′*)—*π*_obs_—*τ*_2_—Echo) in a Q‐Band spectrometer at 50 K. The echo amplitude was recorded as a function of the dipolar evolution time *t*. The pump and observer pulses were positioned on the global maximum and close to the most intense local maximum (shifted by 70 MHz) of the spectrum, respectively. Data were analyzed with the help of DeerAnalysis.^34^ The rotamer library was built as recently described.^32^ Further experimental details are given in the Supporting Information.

## Conflict of interest

*The authors declare no conflict of interest*.

## Supporting information

As a service to our authors and readers, this journal provides supporting information supplied by the authors. Such materials are peer reviewed and may be re‐organized for online delivery, but are not copy‐edited or typeset. Technical support issues arising from supporting information (other than missing files) should be addressed to the authors.

Supplementary
