# Supplementary material for: Isoindoline‐Based Nitroxides as Bioresistant Spin Labels for Protein Labeling through Cysteines and Alkyne‐Bearing Noncanonical Amino Acids
Source: Chembiochem. 2019 Dec 6;21(7):958–62. doi: 10.1002/cbic.201900537 (PMC7187341; doi:10.1002/cbic.201900537)
Supplement: Supplementary file 1 — Supplementary [file CBIC-21-958-s001.pdf]

CHEMBIOCHEM

Supporting Information

Isoindoline-Based Nitroxides as Bioresistant Spin Labels for Protein Labeling through Cysteines and Alkyne-Bearing Noncanonical Amino Acids

Theresa Sophie Braun,[a, b] Pia Widder,[a, b] Uwe Osswald,[a] Lina Groß,[a] Lara Williams,[a] Moritz Schmidt,[a] Irina Helmle,[a, d] Daniel Summerer,*[c] and Malte Drescher*[a, b]

cbic_201900537_sm_miscellaneous_information.pdf

Table of Contents

A. Synthetic procedures S2

General procedures S2

Experimental procedures S2

B. Characterization S6

NMR S6

LC-MS S10

C. Molecular biology S12

Site-directed mutagenesis S12

Transformation in *E. coli* S12

Protein expression and purification S12

Site-directed spin-labeling (SDSL) S12

Maleimide coupling S12

Copper(I)-catalyzed azide-alkyne cycloaddition (CuAAC) S13

D. Bioresistancy assay S14

Preparation of sodium ascorbate assay S14

Preparation of bacterial lysate assay S14

Preparation of HEK lysate assay S14

X-Band CW EPR measurement S14

X-Band CW EPR data analysis S14

E. Distance Determination S15

Sample preparation S15

DEER measurement S15

Data analysis S15

Rotamer library S15

F. Supplementary figures S16

G. List of Abbreviations S19

H. References S20

A. Synthetic procedures

General procedures

Technical solvents were distilled prior to use. Dry solvents were purchased from Sigma-Aldrich and Acros. Reagents were purchased from Sigma-Aldrich, Acros, Merck and TCI and used without further purification. All reactions were monitored by TLC on silica gel 60 F254 coated aluminum sheets (*Merck*) with detection by UV light (λ = 254 nm, 366 nm). Iodine and basic aqueous potassium permanganate solution, followed by gentle heating, was additionally used for visualization. Preparative flash chromatography (FC) was performed on silica gel 60 M from *Macherey-Nagel* with solvent systems as specified. High-resolution mass spectra (HRMS) were recorded on a Bruker micrOTOF II with electrospray ionization in acetonitrile and water with 0.1 % TFA. Analytical high-performance liquid chromatography-mass spectrometry (LC-MS) was performed on a LC/MS-2020 system from *Shimadzu* (high pressure pumps LC-20AD, auto sampler SIL-20AT HAT, column oven CTO-20AC, UV-Vis detector SPD-20A, communication bus module CBM-20A, LC/MS-2020 ESI detector and LC-MS solution software) with a Kinetex 2.6 µm C18 100 Å column (150 x 4.60 mm, flow 0.4 mL min^{-1}) from *Phenomenex* as stationary phase. A binary gradient of acetonitrile in water with 0.1% formic acid was used as mobile phase. ^{1}H NMR spectra were recorded on a Bruker Avance III 400 instrument. Chemical shifts are reported relative to solvent signals ($CDCl_3$: δH = 7.26 ppm).

Experimental procedures

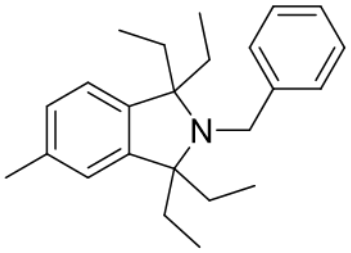

1

Amine **1** was synthesized as previously described.[1]

^{1}H-NMR (400 MHz, $CDCl_3$): δ = 7.48 (d, *J* = 7.1 Hz, 2 H, ArH), 7.36-7.22 (m, 3 H, ArH), 7.05 (d, *J* = 7.7 Hz, 1 H, ArH), 6.97 (d, *J* = 7.7 Hz, 1 H, ArH), 6.89 (s, 1 H, ArH) 4.03 (s, 2 H, CH_2), 2.40 (s, 3 H, CH_3), 1.94 (dsex, *J* = 7.4 Hz, 2.0 Hz, 4 H, 2 x CH_2), 1.63-1.49 (m, 4 H, 2 x CH_2), 0.81 (dt, *J* = 7.4 Hz; 3.1 Hz, 12 H, 4 x CH_3) ppm.

^{13}C-NMR (101 MHz, $CDCl_3$): δ = (144.9, 142.7, 141.9, 135.2, 129.4, 127.9, 126.7, 126.6, 124.1, 123.3, 71.4, 71.2, 46.9, 30.52, 30.50, 21.6, 9.8, 9.7) ppm.

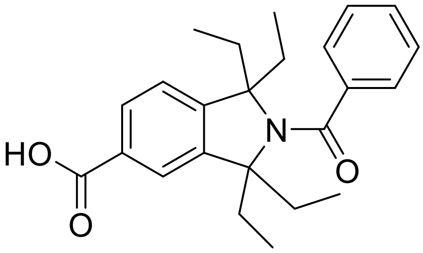

2

2-Benzyl-5-methyl-1,1,3,3-tetraethylisoindoline **1** (4.1 g, 12.1 mmol, 1 eq) and sodium hydroxide (2.8 g, 70.2 mmol, 5.8 eq) were suspended in a mixture of pyridine (30 mL) and water (46 mL) and potassium permanganate (33.9 g, 214.3 mmol, 17.7 eq) was added portion wise. The mixture was refluxed at 120 °C for 48 h and then cooled to room temperature. Ethanol (50 mL) was added and the reaction mixture was filtered. The filter was washed three times with ethanol (50 mL) and the solvent was evaporated. The residual yellow oil was diluted with water (50 mL) and acidified with hydrochloric acid to pH 1. A white precipitate was formed, dissolved in

diethyl ether, the aqueous phase extracted with diethyl ether (5 x 100 mL), dried over $MgSO_4$, and the solvent removed under reduced pressure to obtain alcohol **2** as an off-white solid (3.7 g, 9.7 mmol, 80 %). HRMS: calcd. for $C_{18}H_{29}NO_4S^{\cdot}$: 380.2226 $[M+H]^+$, 380.2213 found.

^{1}H NMR (400 MHz, MeOD): δ = 8.03 (d, *J* = 7.9 Hz, 2 H, ArH), 7.57-7.30 (m, 6 H, ArH), 2.49 (bs, 4 H, 2 x CH_2), 1.99 (bs, 2 H, CH_2), 1.64 (bs, 2 H, CH_2 0.92 (bs, 6 H, 2 x CH_3), 0.75 (bs, 6 H, 2 x CH_3)ppm.

^{13}C NMR (101 MHz, MeOD): δ = (173.6, 169.4, 140.5, 131.6, 130.2, 130.0, 129.1, 127.0, 35.0, 32.3, 10.2) ppm.

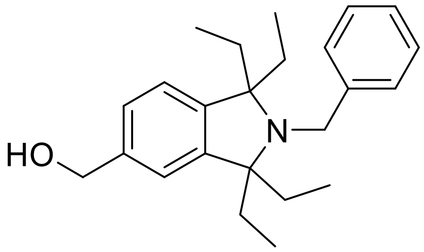

3

To a solution of 2-Benzoyl-1,1,3,3-tetraethylisoinoline carboxylic acid (3.7 g, 9.7 mmol, 1 eq, **2**) in THF (100 mL) at 0° C lithium aluminum hydride (3.3 g, 87.5 mmol, 9 eq) was added in small portions and the solution was refluxed for 48 h. After cooling to RT, the reaction was quenched with water (20 mL) and stirred for another 20 min, before adding ethyl acetate (50 mL). The suspension was filtered and extracted with ethyl acetate (3 x 50 mL). After drying over $MgSO_4$, the solvents were removed to yield a reddish oil. The crude product was purified by column chromatography (3:1 Hexane : Ethyl acetate) to obtain alcohol **3** (3.4 g, 9.6 mmol, 99 %) as a reddish oil. TLC: R_f = 0.14 (PE/DCM 7:3, UV, Iodine); HRMS: calcd. for $C_{18}H_{29}NO_4S^{\cdot}$: 352.2640 $[M+H]^+$, 352.2621 found.

^{1}H NMR (400 MHz, $CDCl_3$): δ = 7.44 (d, *J* = 7.1 Hz, 2 H, ArH), 7.32-7.17 (m, 4 H, ArH), 7.11-6.95 (m, 2 H, ArH), 4.69 (s, 2 H, CH_2), 4.00 (s, 2 H, CH_2), 2.00-1.80 (m, 4 H, 2 x CH_2), 1.66-1.46 (m, 4 H, 2 x CH_2), 0.76 (td, *J* = 7.4, 2.0 Hz, 12 H, 4 x CH_3) ppm.

^{13}C-NMR (101 MHz, $CDCl_3$): δ = (145.3, 144.4, 142.4, 138.6, 129.4, 127. 9, 126.7, 124.9, 123.6, 122.2, 71.4, 65.8, 46.9, 30.5, 9.8, 9.7) ppm.

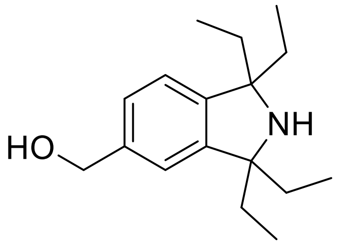

4

To a solution of **3** (1.389 g, 3.95 mmol) in AcOH (20 mL), Pd on charcoal (120 mg) was added under argon atmosphere. Then, it was degassed and hydrogen atmosphere was applied before it was stirred overnight. The suspension was filtered through a methanol washed pad of celite and the pH of the collected filtrate was basified with sat. $NaHCO_3$ and 1 M NaOH. The cloudy suspension was then extracted with ethyl acetate (3 x 100 mL). Organic phases were pooled, dried over $MgSO_4$ and all solvents were removed to obtain the product as a crude slight orange oil. The crude oil was purified by column chromatography (EE:PE 1:2 then EE:PE 2:1) to yield a yellow oil (615 mg, 47 %). TLC: R_f = 0.11 (DCM/MeOH 25:1, $KMnO_4$); HRMS: calcd. for $C_{18}H_{29}NO_4S^{\cdot}$: 262.2171 $[M+H]^+$, 362.2156 found.

^{1}H NMR (400 MHz, $CDCl_3$): δ = 7.22 (d, *J* = 7.8 Hz, 1 H, ArH), 7.09-7.05 (m, 2 H, ArH), 4.69 (s, 2 H, CH_2), 1.88-.61 (m, 8 H, 4 x CH_2), 0.88 (td, *J* = 7.5, 2.1 Hz, 12 H, 4 x CH_3) ppm.

^{13}C-NMR (101 MHz, $CDCl_3$): δ = (142.7, 141.8, 139.1, 126.0, 122.7, 121.3, 65.4, 74.0, 30.9, 30.4, 29.7, 29.4, 8.9, 9.1, 9.2, 9.5) ppm.

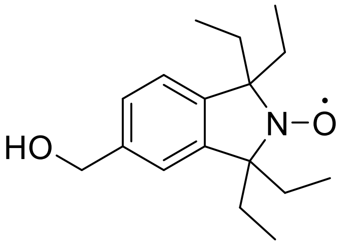

5

Compound **4** (602 mg, 2.3 mmol) was dissolved in anhydrous DCM (18 mL) under N_2 atmosphere and cooled to 0 °C. mCPBA (1.14 g, 4.6 mmol) and $MgSO_4$ were degassed and DCM was added (18 mL) under N_2 atmosphere. To extract the pure mCPBA, the solution was filtered with a filter canula and added dropwise to the cooled solution of **4**. After 10 min, the addition was complete, and the mixture was stirred at 0 °C for 20 h. Ether (180 mL) was then added and the mixture washed with sat. $NaHCO_3$ solution (2x 20 mL) and brine (20 mL). Then, all volatile compounds were removed yielding crude yellow crystals. The compound was purified by column chromatography (light petroleum/ethyl acetate 4:1) to give a yellow oil (486 mg, 76 %). TLC (PE/EE 4:1, UV, $KMnO_4$): $R_f = 0.23$; HRMS: calcd. for $C_{18}H_{29}NO_4S^{\cdot}$: 277.2042 $[M{+}H]^+$, 277.1987 found.

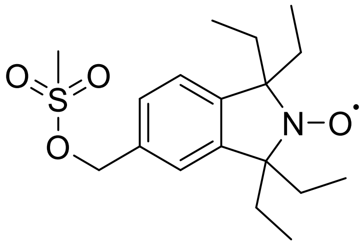

6

To a solution of alcohol **5** (50 mg; 181 µmol) in dry DCM (6 mL) at 0 °C, NEt_3 (0.06 µL; 434 µmol) was added followed by mesylchloride (0.15 mL; 1.99 mmol). The mixture was stirred for 3 h at 0 °C before it was allowed to warm to RT. After quenching with sat. $NaHCO_3$ (10 mL), the phases were separated and the aqueous phase extracted with DCM (2 x 10 mL). The combined organic phases were dried over $MgSO_4$ followed by removal of all volatile compounds to yield a crude yellow liquid, which was purified by column chromatography (light petroleum (PET)/EtOAc 4:1) to yield mesylate **6** (56.5 mg; 88 %) as a yellow oil. $R_f = 0.22$ (PET/EtOAc 4:1); LC-MS: $R_t = 8.741$ min (60–100% MeCN in water over 10 min); HRMS: calcd. for $C_{18}H_{29}NO_4S^{\cdot}$: 355.1817 $[M{+}H]^+$, 355.1768 found.

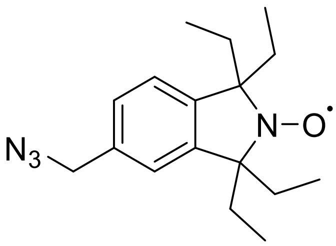

7

Sodium azide (205 mg; 3.16 mmol) was added to a solution of mesylate **6** (56 mg; 158 µmol) in dry DMF (6 mL) and the solution was stirred at RT for 20 h. The reaction was diluted with EtOAc and washed with brine (2 x 20 mL). The organic layer was dried with $MgSO_4$ and the solvents were removed to yield a crude yellow oil which was purified by column chromatography (PET/EtOAc 25:1) yielding azide **7** (37.5 mg; 79%) as a yellow oil. $R_f = 0.68$ (PET/EtOAc 4:1); LC-MS: $R_t = 7.908$ min (80–100% MeCN in water over 10 min); HRMS (ESI-TOF) m/z: $[M + H]^+$ calcd for $C_{17}H_{26}N_4O^{\cdot}$ 302.2107; found 302.2054.

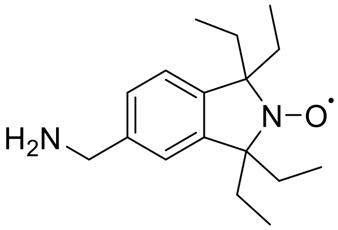

8

To a cooled solution of azide **7** (32.5 mg; 108 µmol) in dry THF (5 mL) under N_2 atmosphere, triphenylphosphine (85 mg; 323 µmol) was added. After 30 min of stirring ammonia solution (2 mL, 25 %) was added and stirring was continued for 21 h. Water (5 mL) and DCM (30 mL) were then added to the mixture, and the aqueous phase was extracted with DCM (2 x 5 mL). The organic phase was dried over $MgSO_4$ and all solvents removed to yield a crude yellow oil. It was purified by column chromatography (DCM/MeOH 9:1) to obtain amine **8** (29.8 mg, quant.), which was used without further characterization for the next step. R_f = 0.31 (DCM/MeOH 9:1).

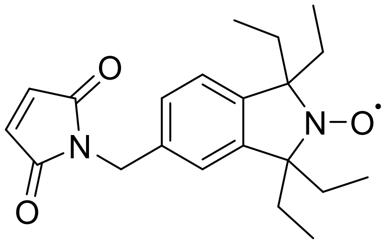

9

Amine **8** (29.8 mg; 108 µmol) and maleic anhydride (13.8 mg; 141 µmol) were dissolved in dry THF (2.5 mL) and was stirred for 50 min before the solvents were removed under reduced pressure. Anhydrous sodium acetate (47.6 mg; 580 µmol) was added to the residue before it was dissolved in acetic anhydride (2 mL) and heated to 74 °C for 1.5 h. After removal of all volatile compounds the crude black residue was purified by column chromatography (PET/EtOAc 4:1) yielding maleimide **9** (24.5 mg; 64 %) as a yellow oil. R_f = 0.23 (PET/EtOAc 4:1); LC-MS: R_t = 9.641 min (60–100% MeCN in water over 10 min); HRMS (ESI-TOF) m/z: $[M + H]^+$ calcd for $C_{21}H_{28}N_2O_3^{\cdot}$ 356.2100 ; found 356.2094.

B. Characterization

NMR

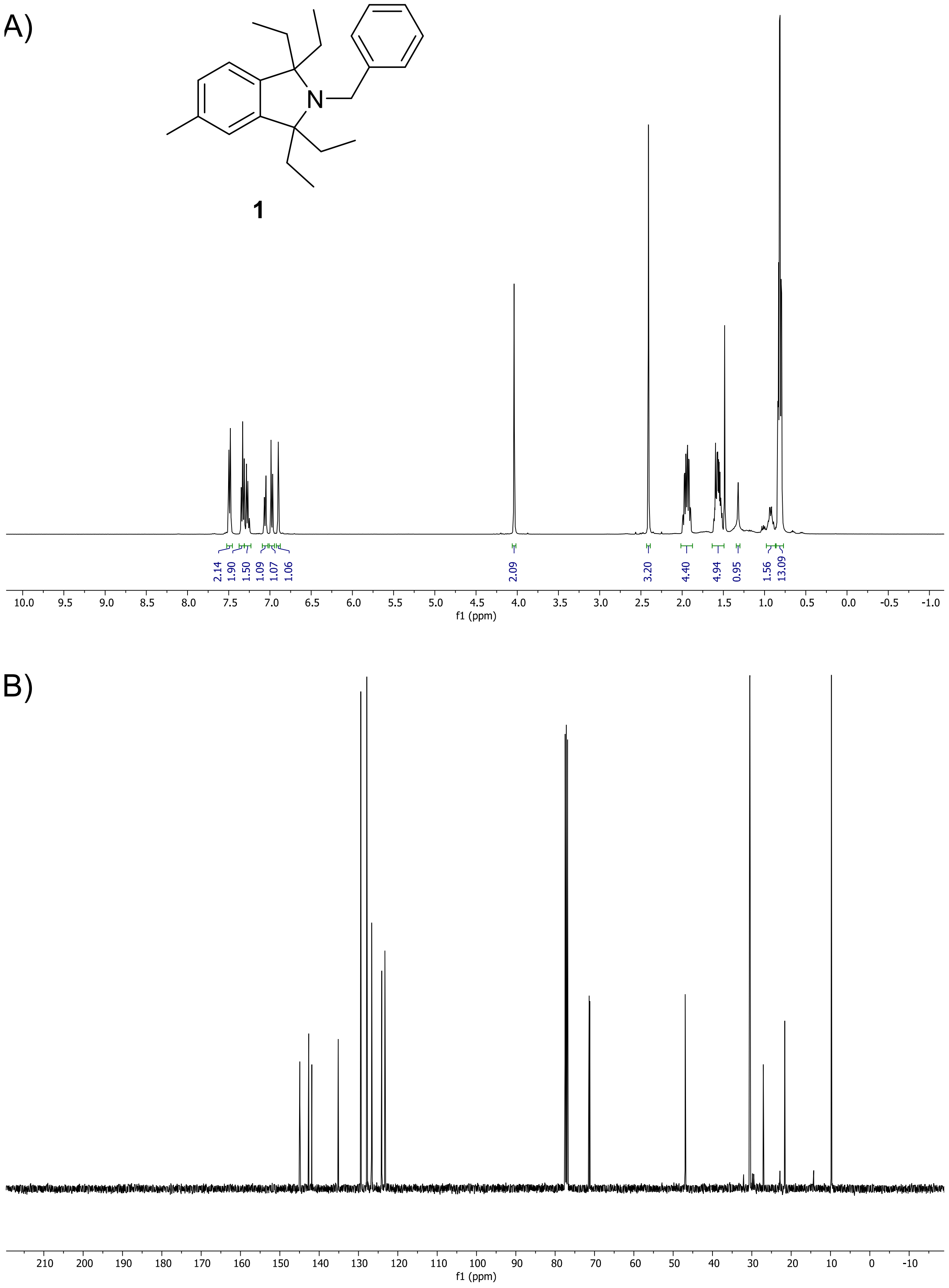

Figure S1. (**A**) ^{1}H NMR (400 MHz, $CDCl_3$) and (**B**) ^{13}C NMR (101 MHz, $CDCl_3$) spectra of **1**.

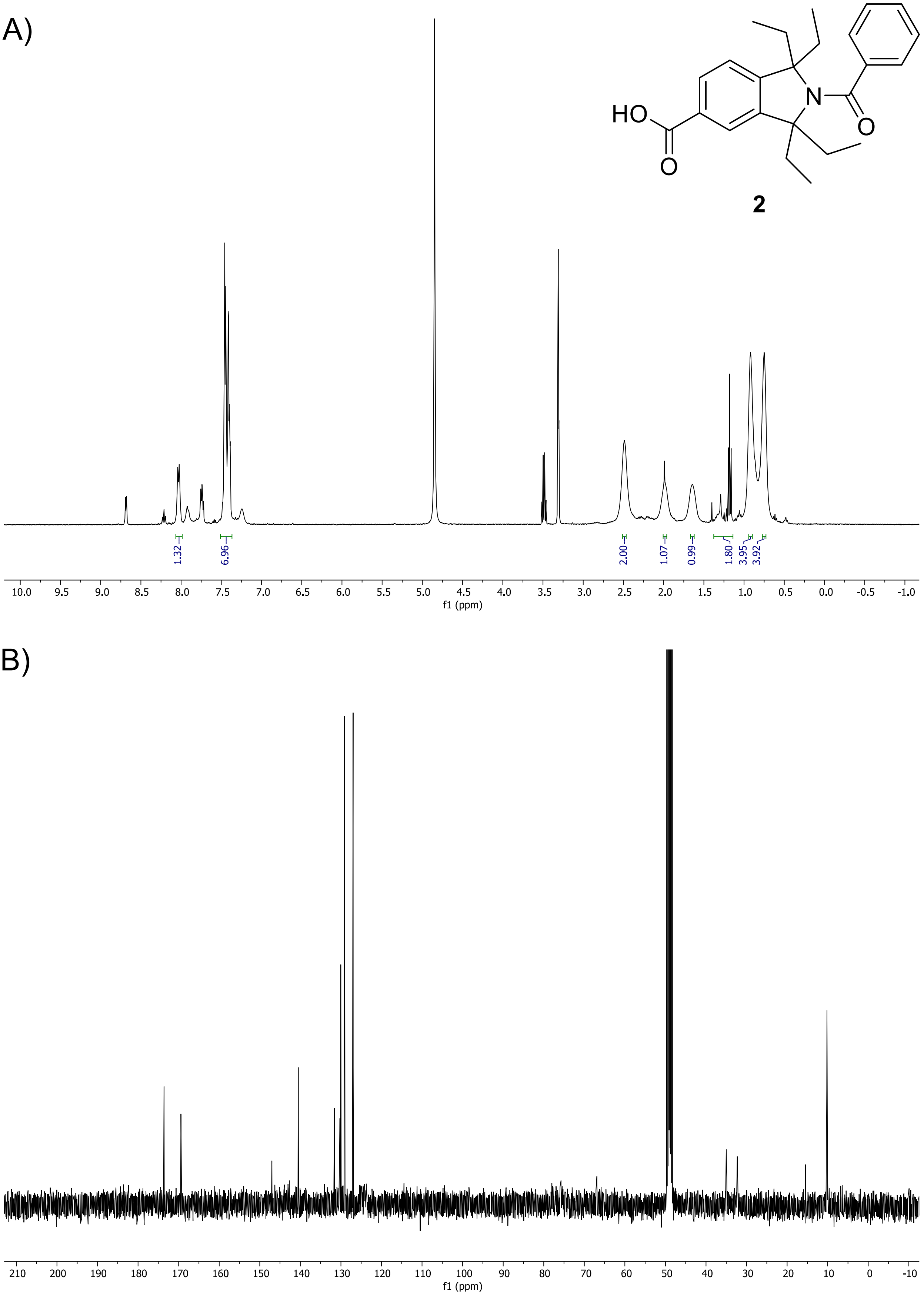

Figure S2. (**A**) ^{1}H NMR (400 MHz, MeOD) and (**B**) ^{13}C NMR (101 MHz, MeOD) spectra of **2**.

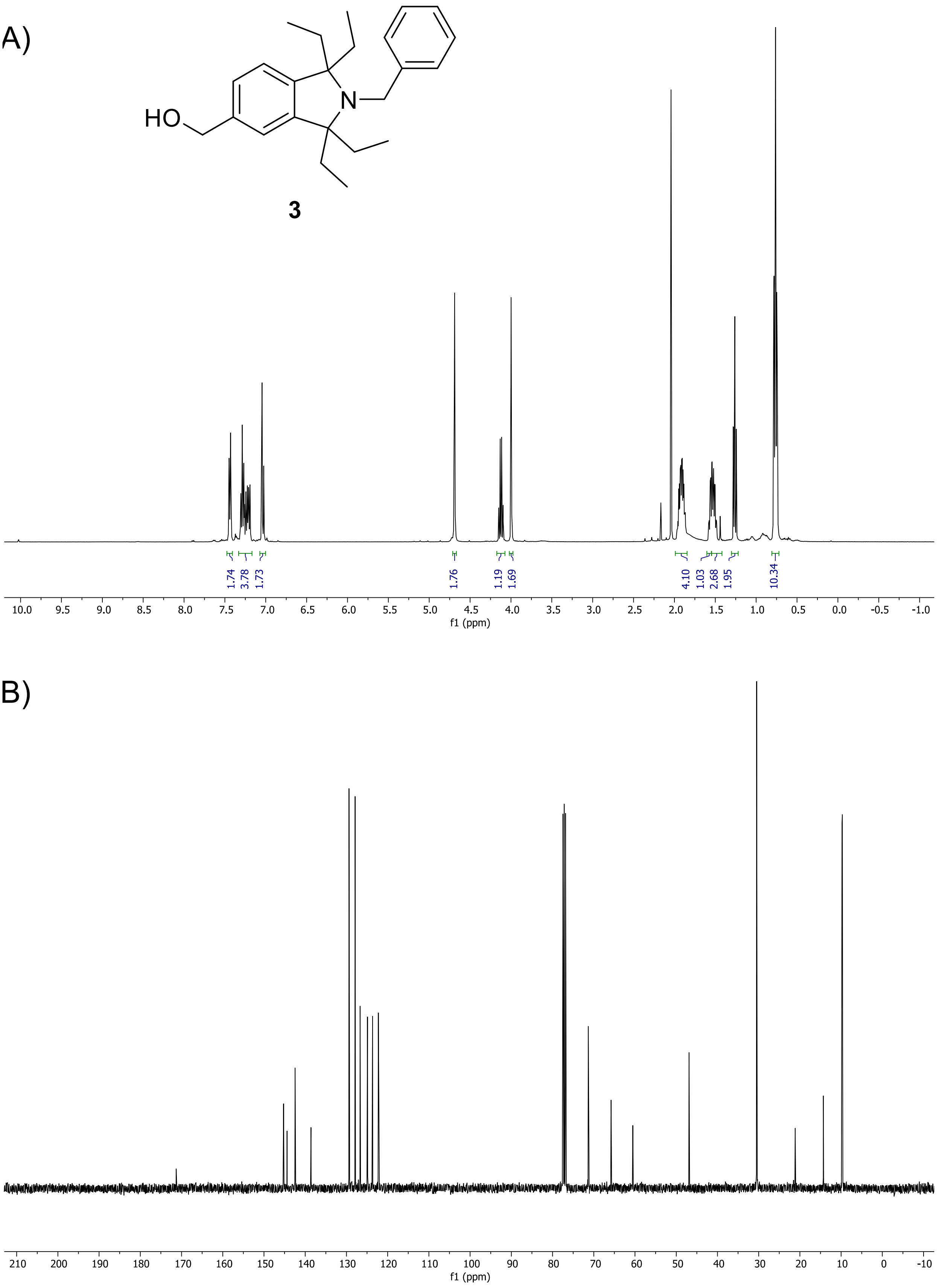

Figure S3. (**A**) ^{1}H NMR (400 MHz, $CDCl_3$) and (**B**) ^{13}C NMR (101 MHz, $CDCl_3$) spectra of **3**. Peaks at 14.3, 21.2, and 171.3 ppm result from remaining EE.

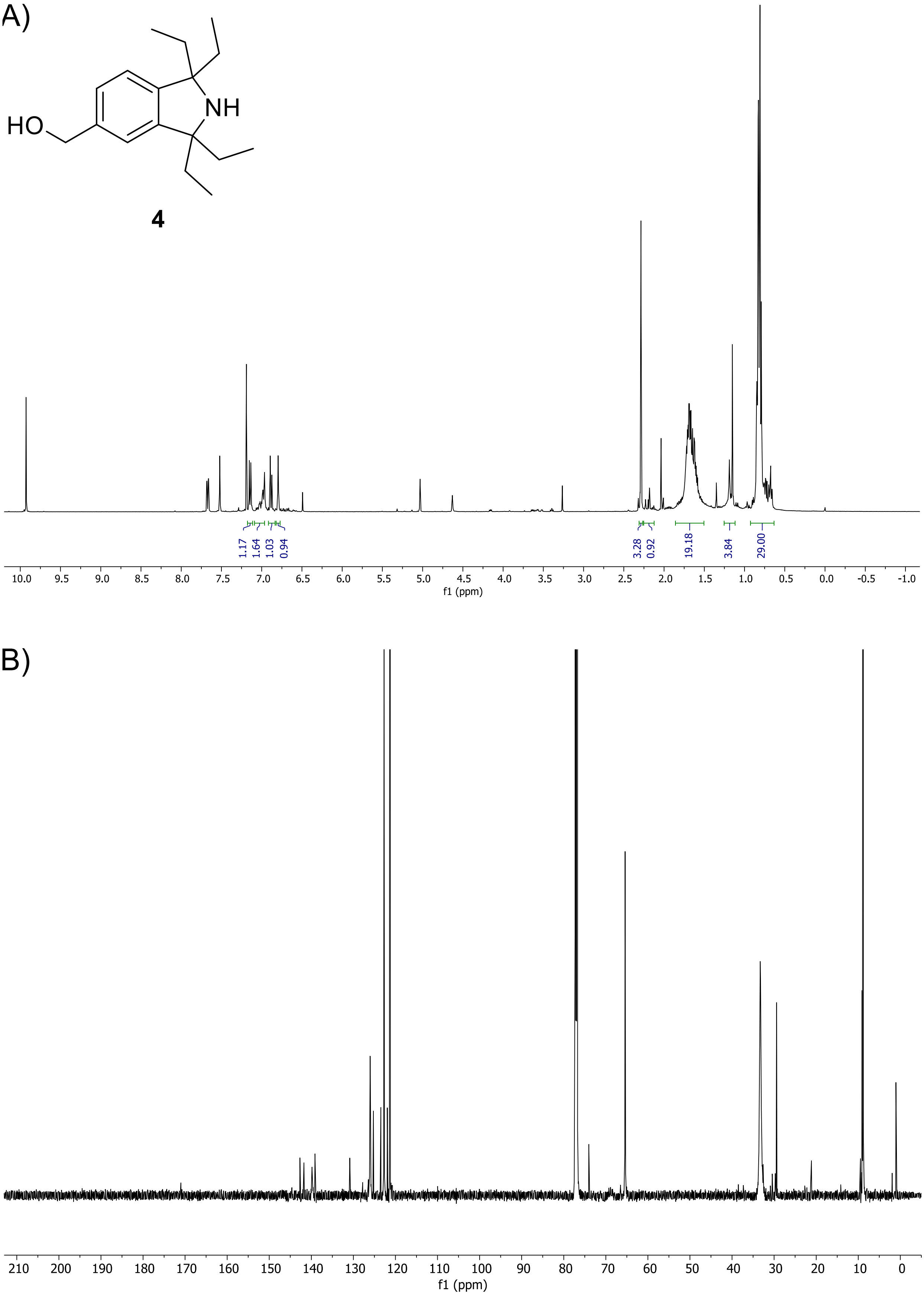

Figure S4. (**A**) ^{1}H NMR (400 MHz, $CDCl_3$) and (**B**) ^{13}C NMR (101 MHz, $CDCl_3$) spectra of **4**.

LC-MS

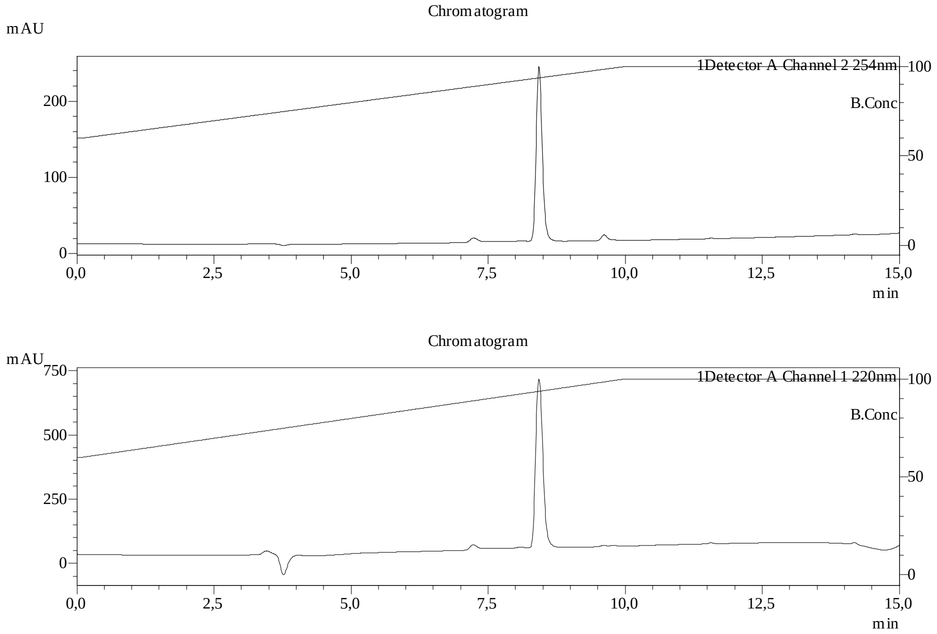

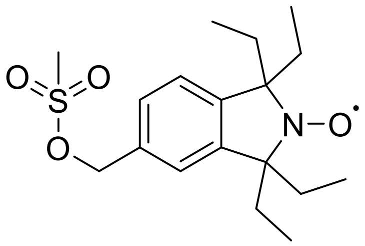

6

Figure S5. LC-MS chromatograms of **6**. 60-100 10 min

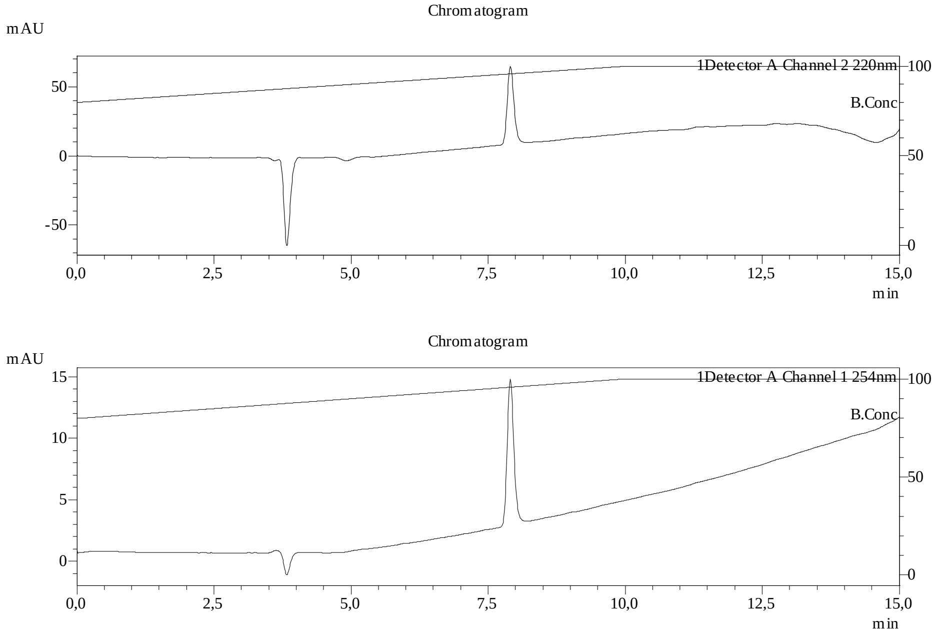

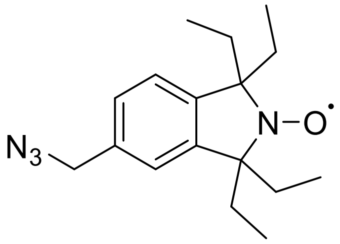

7

Figure S6. LC-MS chromatograms of **7**. 80-100 10 min

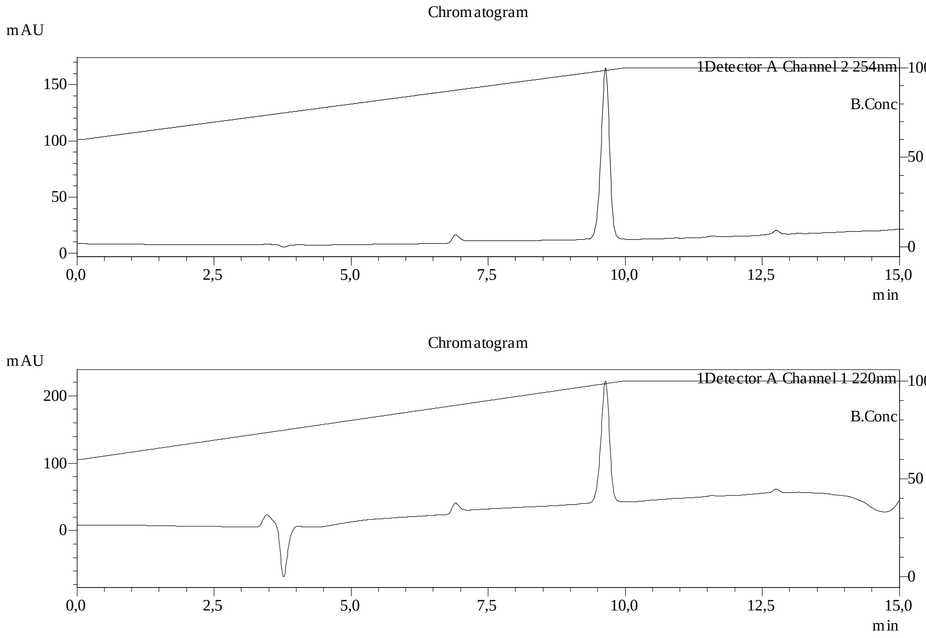

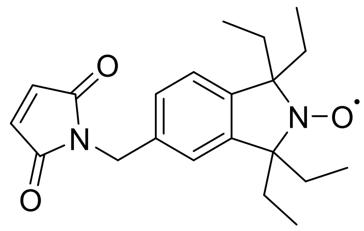

9

Figure S7. LC-MS chromatograms of **9**. 60-100 10 min

C. Molecular biology

Site-directed mutagenesis

Plasmids pBAD_TRX_His6_C33S_C36S (TRX wt*), pBAD_TRX_His6_C33S_C36S_D14C_R74C (TRX D14C R74C), pBAD_TRX_His6_C33S_C36S_D14C_G34C (TRX D14C G34C), pBAD_TRX_His6_C33S_C36S_D14stop_G34stop (TRX D14TAG G34TAG) were generated by site-directed mutagenesis (QuikChange II site-directed mutagenesis kit, Agilent) on basis of pBAD_TRX_His6[2] (TRX wt) as recently described[3].

Transformation in *E. coli*

Chemically competent BL21-Gold (DE3) *E. coli* cells were thawed on ice, mixed with the respective plasmid, and incubated on ice for 30 min, before being heat-shocked at 42 °C for 1 min with subsequent incubation on ice (2 min) and being rescued in pre-warmed (37 °C) Super Optimal Broth with carbolite repression (S.O.C.) medium for 1 h at 37 °C and 140 rpm. Transformed cells were grown on LB-agar plates (Lennox) containing 50 µg/mL carbenicillin at 37 °C and 180 rpm overnight. For glycerol stock creation, a single colony was picked for inoculation of 10 mL LB-medium containing 50 µg/mL carbenicillin, grown overnight at 37 °C and 180 rpm, mixed with 50 % (v/v) glycerol, shock-frozen in liquid nitrogen and stored at -80 °C.

Plasmid pBAD_TRX_His6_C33S_C36S_D14stop_G34stop (TRX D14TAG G34TAG) was co-transformed with plasmid pEVOL_pCNF_YRS encoding a polyspecific *Methanocaldococcus jannaschii* $tRNA^{Tyr}$(CUA)/tyrosyl-tRNA-synthetase (YRS) pair evolved for the genetic encoding of para-cyano-L-phenylalanine, *p*CNF, as provided by the Schultz lab.[4] The transformation was performed as described above with addition of 34 µg/mL chloramphenicol to all LB-media.

Protein expression and purification

1 L LB medium containing carbenicillin (50 µg/mL) were inoculated with 1% overnight culture of BL21-Gold(DE3) *E. coli* (Agilent) containing plasmid pBAD_TRX_His6_C33S_C36S (TRX wt*), pBAD_TRX_His6_C33S_C36S_D14C_R74C (TRX D14C R74C), or pBAD_TRX_His6_C33S_C36S_D14C_G34C (TRX D14C G34C). For the expression of proteins containing *p*ENF, bacteria that were cotransfected with plasmids pEVOL_pCNF_YRS and pBAD_TRX_His6_C33S_C36S_D14C_G34C (TRX D14C G34C) were used, and chloramphenicol (34 µg/mL) and carbenicillin (50 µg/mL) were added to the medium. At OD_{600} = 0.8 protein expression was induced in presence of 2 g L-Arabinose. After 5 h of incubation at 37 °C and 180 rpm shaking, expression was stopped via centrifugation. Lysis was performed in 25 mL bacterial protein extraction reagent (B-PER ™, Thermo Fisher Scientific), in presence of 1 mM phenylmethylsulfonylfluoride (PMSF, protease-Inhibitor) and 1 mM dithiothreitol (DTT) with a Q700 SONICATOR (QSONICA, equipped with a 1.6 mm tip probe, 2 min cycling with 1 s pulse-on and 1 s pulse-off, amplitude 10). Protein extraction was performed with Ni-NTA (Nickel- nitrilotriacetic acid) Agarose Resin (Thermo Fisher Scientific) using the high affinity of the C-terminal 6xHis Tag of thioredoxin. Washing was performed with 50 mL Qiagen Lysis buffer (50 mM NaH_2PO_4, 300 mM NaCl, 10 mM imidazole, pH8) containing DTT (1 mM) in total. After two elution steps with each 500 µL Qiagen Lysis buffer (50 mM NaH_2PO_4, 300 mM NaCl, 500 mM imidazole, pH8), the protein was dialyzed against the target buffer (Tris, 150 mM NaCl, pH 7.4 or PBS, pH 7.5) and concentration measured at a BioPhotometer® D30 (Eppendorf) at 280 nm. Reduction stability in *E. coli* lysate was investigated in PBS, linker stability in deuterated PBS.

Site-directed spin-labeling (SDSL)

Maleimide coupling

If not stated differently, cysteine mutants of thioredoxin were linked to M-TEIO as follows. 100 µL protein (500 µM) were mixed with 2 µL tris(2-carboxyethyl)phosphine (TCEP) (250 mM), incubated on ice for 1 h, and subsequently TCEP was removed with the use of 2x500 µL Zeba desalting columns (7K MWCO) that were equilibrated with 2x500 µL buffer (Tris-HCl, 150 mM NaCl, pH 7.4). 10 equivalents M-TEIO were added and the labeling reaction was performed in a 1.5 mL reaction tube (Eppendorf) at 4 °C, stirring with a stir bar for 1-4 days. The remaining label was removed using a combination of Ni-NTA-chromatography, PD-10 columns and size exclusion spin filters. The protein was bound to 200 µL Ni-NTA agarose resin (Thermo Fisher Scientific) on ice for 1 h and washed 8-10 times with buffer (Tris-HCl, 150 mM NaCl, pH 7.4) containing 20 % dimethyl sulfoxide (DMSO). Elution was performed four times after 20 min incubation on ice with 50 µL Qiagen Lysis buffer (50 mM NaH_2PO_4, 300 mM NaCl, 500 mM imidazole, pH 8) each. Subsequently,

2x500 µL PD-10 columns that were preincubated with 500 µL Tris 150 mM NaCl were used for further elimination of remaining label. Additionally, Amicon Ultra centrifugal filters with 3K MWCO (Millipore) were used for final purification and buffer exchange for deuterated solutions.

Copper(I)-catalyzed azide-alkyne cycloaddition (CuAAC)

The labeling reaction was performed as previously described.[3] Briefly, copper(II)-sulfate ($CuSO_4$) and the ligand 2-(4-((bis((1-(tert-butyl)-1H-1,2,3-triazol-4-yl)methyl)amino)methyl)-1H-1,2,3-triazol-1-yl) acetic acid (BTTAA)[5] were mixed in water in a 1:3 ratio. Ascorbic acid was added in a 1:1 ratio to copper to reduce Cu(II) ions into the catalytically active Cu(I) species. The labeling reagents were then diluted with PBS buffer pH 7.5 and protein, as well as 100 mM Az-TEIO in DMSO, were added, resulting in a final concentration of 1 mM copper(II)-sulfate, 3 mM BTTAA, 1 mM sodium ascorbate, 50 µM protein, and 1 mM spin-label. The CuAAC reaction took place at 25°C for 1 h and 800 rpm in an Eppendorf ThermoMixer C. Afterward, excess reagents were removed by size-exclusion chromatography *via* spin desalting columns (Zeba ™ spin desalting columns, 7K MWCO, 2 mL column material, Thermo Fisher Scientific). Additional washing steps were performed *via* ultrafiltration in centrifugal filter units (Amicon ultra-0.5 mL centrifugal filters, 3.5 K MWCO, Merck; 15 min, 12000 rpm, 4 °C) to remove excess reagents and concentrate the protein sample in the process. Protein samples were washed 4 x with 400 µL PBS buffer containing 1 mM EDTA to remove remaining copper ions, followed by 6 x 400 µL 1.25 x D_2O-PBS (samples prepared for CW EPR and subsequent DEER measurements).

D. Bioresistancy assay

Preparation of sodium ascorbate assay

L(+)-Ascorbic acid (Carl Roth) was dissolved in water and pH adjusted with sodium hydroxide to yield a stock solution of 200 mM, pH 7, of which 1 µL were mixed with 49 µL label in DMSO/H_2O (50 %, v/v) to a starting spin concentration of 200 µM.

Preparation of bacterial lysate assay

20 mL LB-medium were inoculated with BL21-Gold(DE3) *E. coli* and grown overnight at 37 °C and 180 rpm in presence of tetracycline (10 µg/mL). The cells were harvested via centrifugation, resuspended in 1 mL PBS, and lysed via sonication (Q700 SONICATOR equipped with a 1.6 mm tip probe, 2 min cycling with 1 s on and 1 s off, amplitude 10, QSONICA). For stability measurements, 20 µL lysate were mixed with labeled protein (TRX D14C R74C) to a starting spin concentration of 30 µM.

Preparation of HEK lysate assay

Three dishes (10 cm diameter) of HEK293 (Merck, #85120602) were grown in Dulbecco's Modified Eagle Medium (DMEM, Gibco) containing 9 % fetal bovine serum (FBS) and 0.9 % Penicillin-streptomycin (10,000 U/mL) in humidified 5 % (v/v) CO_2 incubators at 37 °C to 100 % confluence, harvested with the use of trypsin, unified, washed with PBS, and resuspended in 500 µL PBS. After sonication (Q700 SONICATOR equipped with a 1.6 mm tip probe, 2 min cycling with 1 s on and 1 s off, amplitude 10, QSONICA), 20 µL lysate were mixed with labeled protein (TRX D14C R74C) to a starting spin concentration of 6 µM.

X-Band CW EPR measurement

All samples were loaded into glass capillaries with 1 mm inner diameter (HIRSCHMANN® ringcaps®). Ascorbate reduction was monitored on an EMXnano benchtop X-band spectrometer (Bruker Biospin) (9.637 GHz) using a microwave power of 3.162 mW, a modulation amplitude of 0.8 G at a modulation frequency of 100 kHz at room temperature. The EPR-signal reduction in lysates was monitored on a MiniScope MS 5000 benchtop X-band spectrometer (magnettech) (9.645 GHz) using a microwave power of 6.310 mW, a modulation amplitude of 0.8 G at a modulation frequency of 100 kHz at room temperature. Spectra were continuously recorded with a sweep time of 60 s and a sweep width of 150 G.

.

X-Band CW EPR data analysis

Spectra were corrected according to the microwave frequency and baseline-corrected. During time-dependent measurements, we did not observe a change of the spectral shape. Therefore, the peak-to-peak intensity of the central line was extracted for every single scan as a measure for nitroxide concentration. The peak-to-peak intensity was set to I_0 for the first measurement directly after sample preparation (t = 0 h). Intensities were plotted against the time and nitroxide stability characterized by the half-life time τ as detected by the time that corresponds to $I = \frac{1}{2} I_0$.

E. Distance Determination

Sample preparation

DEER experiments were performed at a TRX concentration of 34 µM (50 µM spin concentration) for M-TEIO and 90 µM (108 µM spin concentration) for Az-TEIO in the presence of 20 % (v/v) glycerol-d8. Samples were loaded into quartz sample tubes (Fused quartz tubing, Technical Glass Products; 2 mm inner diameter) and shock frozen in liquid nitrogen before measurement.

DEER measurement

The experiments were performed using an ELEXSYS E580 spectrometer (Bruker Biospin) equipped with a Q-band resonator (ER5106QT-2, Bruker Biospin) and a 150 W traveling-wave tube (TWT) amplifier (Applied Systems Engineering, Fort Worth, USA). Samples were held on cryogenic temperatures (50 K) with the EPR Flexline helium recirculation system (CE-FLEX-4K-0110, Bruker Biospin, ColdEdge Technologies) comprising a cold head (expander, SRDK-408D2) and a F-70H compressor (both SHI cryogenics, Tokyo, Japan), controlled by an Oxford Instruments Mercury ITC.

DEER experiments were performed using a four-pulse sequence ($\pi/2_{obs} - \tau_1 - \pi_{obs} - t' - \pi_{pump} - (\tau_1 + \tau_2 - t') - \pi_{obs} - \tau_2$ – Echo). The resonator was overcoupled (Q ≈ 100). In case of M-TEIO pulse lengths were π_{pump} = 17 ns and π_{obs} = 28 ns, for Az-TEIO π_{pump} = 16 ns and π_{obs} = 24 ns. The pulse separation time τ_1 was 400 ns and nuclear modulations were averaged by incrementing τ_1 in 8 steps of 16 ns. The dipolar evolution time τ_2 was 4000 ns. The echo amplitude was recorded as a function of the dipolar evolution time t'. The pump frequency was set to the center of the resonator dip (ν_{pump} = 34 GHz), where the global maximum of the nitroxide EPR spectrum was positioned. The observer frequency was shifted by 70 MHz towards lower frequencies (ν_{obs} = 33.93 GHz) to a local maximum of the nitroxide EPR spectrum. The shot repetition time was 4080 µs. An eight-step phase cycle was used on the pump pulse.[6] A complete DEER experiment was performed as a 2D experiment, where one dimension was the time axis *t'* and the second dimension was the axis of individual scans. The scans were subjected to phase correction individually and subsequently summarized.

Data analysis

DEER data sets were analyzed using the DeerAnalysis[7] 2018 software package for MATLAB. Extraction of the dipolar evolution function was achieved by background correction with a 3-dimensional homogeneous background function followed by model-free Tikhonov regularization. The optimum regularization parameter α ($\alpha_{M\text{-}TEIO}$ = 501, $\alpha_{Az\text{-}TEIO}$ = 794) was determined using the L-curve corner criterion (Figure S10). The resulting distance distributions were validated by the validation tool of the DeerAnalysis 2018 software by varying the background start (11 different values) and noise level (5 different values) during the regularization procedure with a prune level of 1.15. Uncertainties are depicted as shaded areas in the distance distributions *P(r)* shown in Figure 3 normalized such that P_{max} = 1.

Rotamer library

A rotamer library (Figure S12) was generated for *p*ENF-TEIO to enable the prediction of DEER distance distributions.[8] An initial model of Az-TEIO was generated with ChemDraw Professional 16.0 and Chem3D 16.0. DFT calculations with Orca 4.1[9] (!RKS B3LYP/G def2-TZVPP SV(P) TightSCF Opt)[10] were performed with a diamagnetic analog of the spin-label to energy-minimize the label geometry. Five dihedral angles along the label linker were identified (Figure S13 A). An ensemble of 20’000 conformers was generated with MatLab 2019a by randomly varying the dihedral angles and accepting conformers that comply with the criterion $c \leq \exp(-\Delta E/RT)$ with ΔE being the energy difference of the rotamer structure to the reference energy and RT being the thermal energy. The population threshold c was manually assigned to 0.1. Conformer energies were determined by application of a universal force field from the Towhee implementation.[11] Clustering of the ensemble yielded a rotamer library consisting of 4000 population-weighted sets of dihedral angles representing averaged rotamers. MMM 2018_2[12] was used to attach the rotamer library to the PDB structure of thioredoxin (2TRX)[13], remove clashing rotamers and simulate the expected distance distributions for Az-TEIO.

F. Supplementary figures

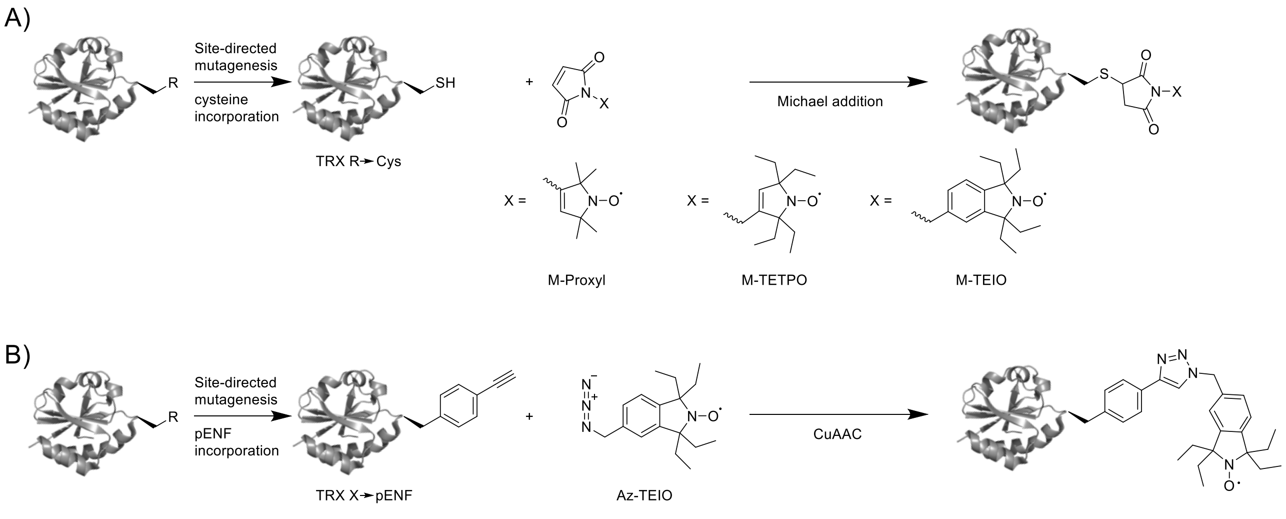

Figure S8. Protein SDSL strategies. (**A**) Michael addition of a maleimide-functionalized labeling reagent with the cysteine variant of the target protein. Maleimide coupling can be performed with M-Proxyl, M-TETPO, and M-TEIO. (**B**) Copper(I)-catalyzed azide-alkyne cycloaddition (CuAAC) between the noncanonical amino acid (ncAA) para-ethynyl-l-phenylalanine (pENF) and Az-TEIO.

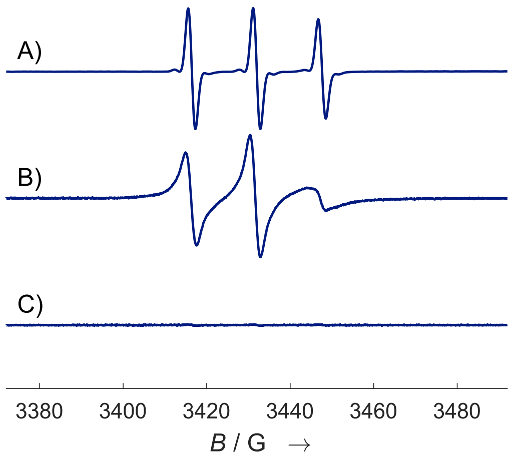

Figure S9. CW EPR spectra. (**A**) M-TEIO (100 µM) in solution features an X-band EPR spectrum in the fast motional regime. (**B**) TRX D14C R74C (100 µM protein, 70 µM spin) features an EPR signal after labeling reaction with M-TEIO. The spectral shape indicates slower nitroxide tumbling compared to **A** suggesting successful labeling. (**C**) No EPR signal was detected for cysteine-free TRX wt* (100 µM protein) upon labeling reaction with M-TEIO, thus proving the cysteine-specificity of the labeling procedure. All spectra were acquired on an EMXnano benchtop X-band spectrometer (Bruker Biospin) (9.637 GHz) using a microwave power of 3.162 mW, a modulation amplitude of 0.8 G at a modulation frequency of 100 kHz at room temperature.

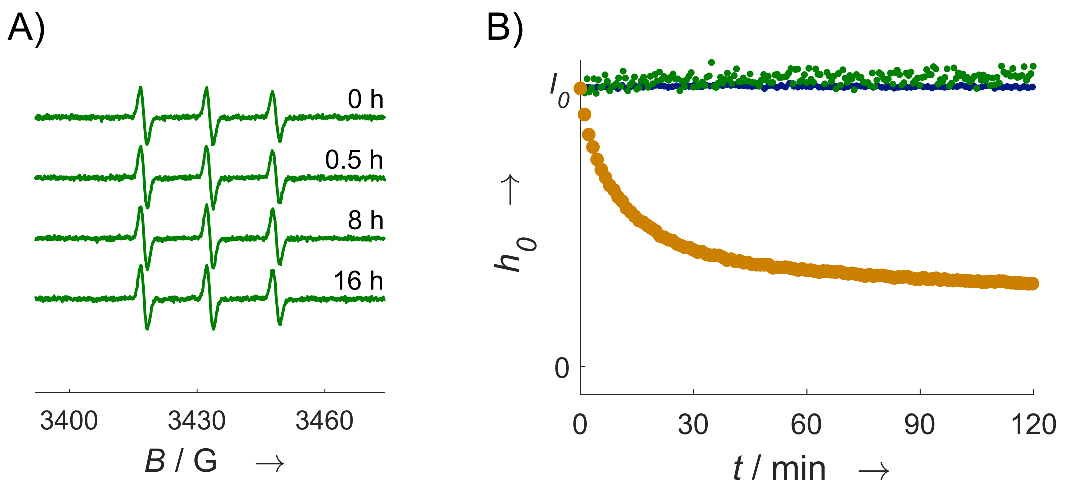

Figure S10. Reduction stability of Az-TEIO (green) in solution with 4 mM ascorbate. (**A**) CW spectra at different time points after mixing. (B) Peak-to-peak intensity was used as a measure for nitroxide stability, set to I_0 for t = 0 h, which corresponds to a nitroxide concentration of 200 µM, and compared to M-TEIO (blue) and M-Proxyl (yellow).

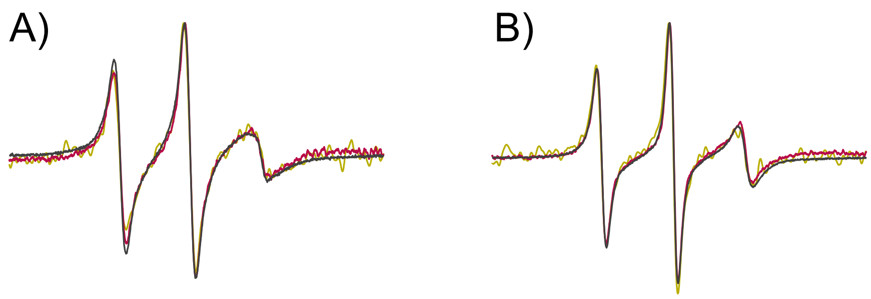

Figure S11. CW spectra of (**A**) M-TEIO or (**B**) M-Proxyl attached to TRX D14C G34C in solution (grey), E. coli lysate (red), or HEK lysate (green).

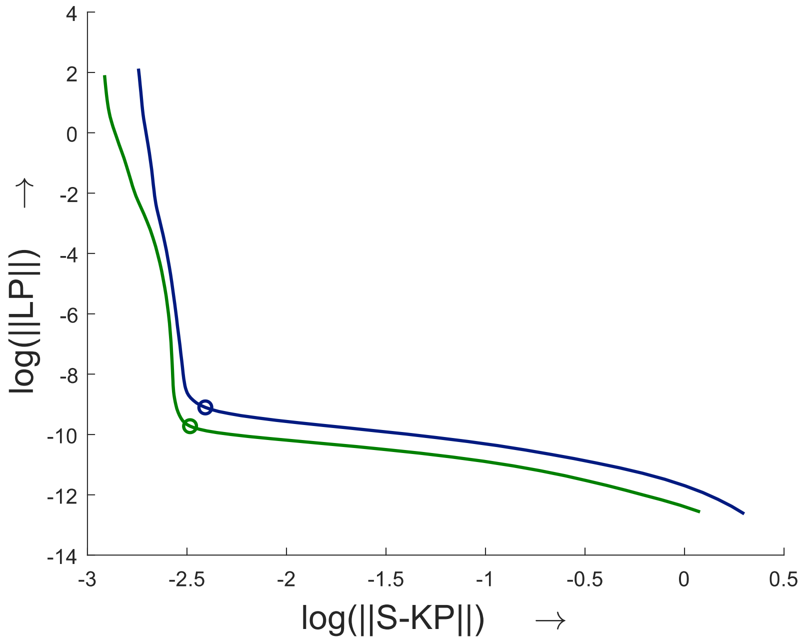

Figure S12. L-curves (roughness of distance distribution plottet against misfit) and α-parameters chosen by the L-curve corner criterion that result in the distance distributions (Figure 3 D). Blue: M-TEIO, green: Az-TEIO.

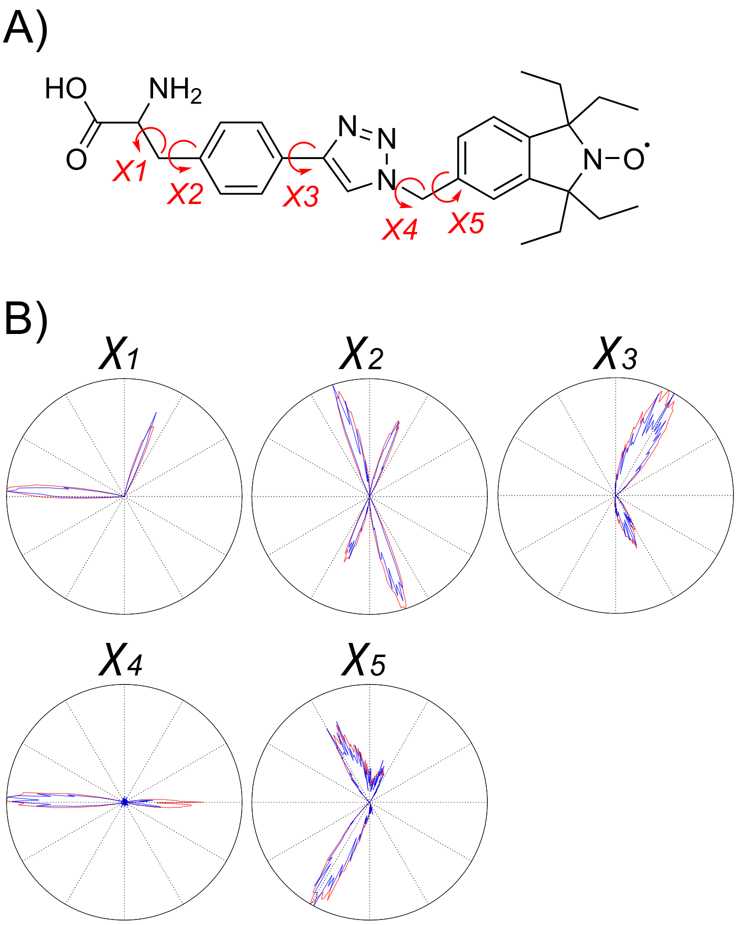

Figure S13. Generation of a rotamer library. (**A**) The structure of Az-TEIO-labeled *p*ENF with the dihedral angles $\chi_{1\text{-}4}$ that were considered in the rotamer library calculation. (**B**) Distributions of the dihedral angles $\chi_{1\text{-}4}$ (red) and superimposed dihedral angles as found in the final rotamer library (blue).

G. List of Abbreviations

**Abbreviation**	**Explanation**
**AcOH**	Acetic acid
**CuAAC**	Copper(I)-catalzyed azide-alkyne cycloaddition
**DCM**	Dichlormethane, CH_2Cl_2
**DEER**	Double electron-electron resonance
E. coli	*Escherichia coli*
**EE**	Ethyl acetate
**EPR**	Electron paramagnetic resonance
**M-TETPO**	Maleimido-Tetraethylpyrrolidinyloxy
**M-Proxyl**	3-Maleimido-2,2,5,5-tetramethyl-1-pyrrolidinyloxy
**mCPBA**	meta-Chloroperoxybenzoic acid
**MeOD**	Deuterated methanol (CD_3OD)
**MTSL**	*S*-(1-oxyl-2,2,5,5-tetramethyl-2,5-dihydro-1H-pyrrol-3-yl)methyl methanesulfonothioate
**ncAA**	Noncanonical amino acid
***p*ENF**	*para*-Ethynylphenylalanine
**RT**	Room temperature
**PE**	Petroleum ether
**SDSL**	Site-directed spin labelling
**SPAAC**	Strain-promoted azide-alkyne cycloaddition
**SPIEDAC**	Strain-promoted inverse-electron-demand Diels-Alder reaction
**TCEP**	Tris-(2-carboxyethyl)phosphin hydrochlorid
**TEIO**	1,1,3,3-**te**tra**e**thyl**i**soindolin-2-yl**o**xyl
**THF**	Tetrahydrofuran
**TLC**	Thin layer chromatography
**TRX**	Thioredoxin
X. laevis	*Xenopus laevis*

H. References

[1] K. E. Fairfull-Smith, F. Brackmann, S. E. Bottle, *Eur J Org Chem* **2009**, *2009*, 1902.
[2] M. J. Schmidt, D. Summerer, *Angew Chem Int Ed Engl* **2013**, *52*, 4690.
[3] P. Widder, F. Berner, D. Summerer, M. Drescher, *ACS Chem Biol* **2019**, *14*, 839.
[4] a) C. C. Liu, P. G. Schultz, *Annu Rev Biochem* **2010**, *79*, 413; b) D. D. Young, T. S. Young, M. Jahnz, I. Ahmad, G. Spraggon, P. G. Schultz, *Biochemistry* **2011**, *50*, 1894; c) K. C. Schultz, L. Supekova, Y. Ryu, J. Xie, R. Perera, P. G. Schultz, *J Am Chem Soc* **2006**, *128*, 13984.
[5] C. Besanceney-Webler, H. Jiang, T. Zheng, L. Feng, D. Soriano del Amo, W. Wang, L. M. Klivansky, F. L. Marlow, Y. Liu, P. Wu, *Angew Chem Int Ed* **2011**, *50*, 8051.
[6] C. E. Tait, S. Stoll, *Phys Chem Chem Phys* **2016**, *18*, 18470.
[7] G. Jeschke, V. Chechik, P. Ionita, A. Godt, H. Zimmermann, J. Banham, C. R. Timmel, D. Hilger, H. Jung, *Appl Magn Reson* **2006**, *30*, 473.
[8] M. Qi, A. Groß, G. Jeschke, A. Godt, M. Drescher, *J Am Chem Soc* **2014**, *136*, 15366.
[9] F. Neese, *Wiley Interdiscip Rev-Comput Mol Sci* **2012**, *2*, 73.
[10] F. Weigend, R. Ahlrichs, *Phys Chem Chem Phys* **2005**, *7*, 3297.
[11] a) M. G. Martin, *Molecular Simulation* **2013**, *39*, 1212; b) A. K. Rappe, C. J. Casewit, K. S. Colwell, W. A. Goddard, W. M. Skiff, *J Am Chem Soc* **1992**, *114*, 10024.
[12] G. Jeschke, *Protein Sci* **2018**, *27*, 76.
[13] S. K. Katti, D. M. LeMaster, H. Eklund, *J Mol Biol* **1990**, *212*, 167.
